# Preliminary antimycobacterial study on selected Turkish plants (Lamiaceae) against *Mycobacterium tuberculosis* and search for some phenolic constituents

**DOI:** 10.1186/1472-6882-13-365

**Published:** 2013-12-21

**Authors:** Tülin Askun, Emmanuel Mouafo Tekwu, Fatih Satil, Seyma Modanlioglu, Hatice Aydeniz

**Affiliations:** 1 Department of Biology, Faculty of Sciences and Arts, University of Balikesir, Cagis Campus, Balikesir 10145, Turkey; 2 Laboratory for Tuberculosis Research (LTR), Biotechnology Centre, University of Yaoundé I, P.O. Box 8936, Yaoundé, Cameroon

**Keywords:** *Mycobacterium tuberculosis*, Antimycobacterial activity, *Satureja aintabensis*, *Thymus sipthorpii,*Lamiaceae, Phenolic, Flavonoid

## Abstract

**Background:**

The global resurgence of tuberculosis is a significant threat. Lamiaceae members have been used in folk remedies for centuries. This study was designed to assess the *in-vitro* antimycobacterial activity of eighteen crude extracts from six plants (Lamiaceae) and to characterize their phenolic and flavonoid compounds.

**Methods:**

Six Turkish medicinal plants of the family Lamiaceae (*Stachys tmolea* Boiss.*, Stachys thirkei* C. Koch, *Ballota acetabulosa* (L.) Benth., *Thymus sipthorpii* Benth., *Satureja aintabensis* P.H. Davis, and *Micromeria juliana* (L.) Benth. ex Reich.) were collected in 2009 – 2010. Dried and crushed plant samples were subjected to sequential extraction with petroleum ether, ethyl acetate, and methanol in order of increasing polarity. A broth microdilution method was employed to screen extracts against four mycobacterial strains of *Mycobacterium tuberculosis*. Phenolic and flavonoid compounds were characterized by liquid chromatography–mass spectrometry.

**Results:**

*S. aintabensis*, *T. sibthorpii*, and *M. juliana* were found to develop considerable activity against the four strains of *M. tuberculosis* with the minimal inhibitory concentrations value of 12.5-100 μg/ml. *S. aintabensis* and *T. sibthorpii* extracts killed *M. tuberculosis* with the minimum bactericidal concentration value of 50–800 μg/ml. On the basis of these prominent antimycobacterial activity, we suggest that they could be a source of natural anti-tuberculosis agents.

**Conclusion:**

*S. aintabensis* and *T. sibthorpii* showed activity by killing *Mycobacteria* strains. The major phenolic compound was rosmarinic for *T. sibthorpii* and *S. aintabensis.* Flavonoids might be “a modal” for the drug design.

## Background

Tuberculosis (TB) is an infectious bacterial disease caused by *Mycobacterium tuberculosis*, which most commonly affects the lungs. It is a well-known disease that has afflicted humans since ancient times. Although tremendous efforts have been made to control TB at global and national levels, approximately one third of the world’s population is infected with *M. tuberculosis*, eight million people develop tuberculosis disease annually, while two million people die and another three million new cases occur each year [1]. The global resurgence of TB and the development of drug resistance, multidrug-resistant (MDR) and extensively drug resistant (XDR) strains present significant threats to TB control. The alarming increase of MDR-TB cases requires the urgent development of new, more effective and safer anti-tuberculosis (anti-TB) drugs.

Lamiaceae members have been used as tea, spice or in folk remedies for centuries. The plant family Lamiaceae has a global distribution, and comprises more than 7200 species across approximately 240 genera [2]. Turkey is regarded as an important gene-center for the plant family Lamiaceae (Labiatae) [3]. The family has 256 species endemic to Turkey, and the rate of endemism in the family is 44.2% [3].

*Stachys L*. is one of the largest genera of the family Lamiaceae, and is used in herbal remedies and is consumed as a tea in Anatolia and Iran. Decoctions or infusions of *Stachys* are applied as tonics to treat skin complaints, or taken internally for stomach disorders [4]. The antibacterial [5], anticancer [6] and antioxidant effects [7] of the genus are described in the literature. The genus *Thymus* (Lamiaceae) has numerous species and varieties and the ratio of endemism in the genus is 53% in Turkey [3]. *Thymus* oil is widely used as an antiseptic agent in many pharmaceutical preparations and as a flavoring agent for many kinds of food products [8]. In Turkey the genus *Ballota* is represented by eleven species and six subspecies, of which ten which are endemic [9]. *Ballota* species have been used in the treatment of wounds and burns, gastrointestinal disorders, as diuretics, in the treatment of hemorrhoids as infusions, as a choleretic and to prevent coughs in Turkish folk medicine [10].

*M. juliana* has several medicinal uses, such as preventing diabetes, tonsillitis, dyspepsia, stomach ulcers, lowering cholesterol, as an anti-spasmodic and preventing bronchitis, common cold, dysmenorrhea, prostate problems and kidney stones [11].

Many members of the genus *Satureja* are well known for their aromatic and medicinal character. They are used as culinary herbs and in folk medicine to treat various ailments, based on their different plant activities. The essential oil isolated from various *Satureja* species possesses certain biological properties, such as antifungal [12], antibacterial [13], anticholinesterase and antioxidant [14], and anti-HIV-1 [15] properties.

Medicinal plants have long been used in traditional healthcare systems to cure various ailments including tuberculosis [16]. Natural plant products have provided an alternative source for the development of antimicrobial drugs [17].

Flavonoids have been recognized as having several biological properties such as being anti-oxidant, anti-allergic and anti-infectious. Some flavonoids show antiviral and antibacterial activities such as quercetin, which is an HIV1-protease inhibitor [18]. Myricetin is also shown to inhibit the growth of vancomycin-resistant enterococci [19].

Numerous examples of interesting secondary metabolites with antimicrobial activity have been isolated from natural sources, indicating that natural products could be a useful field for the discovery of new anti-TB leads. The antimycobacterial properties of medicinal plants are increasingly being reported from different parts of the world, and antimycobacterial activities of members of the Lamiaceae family have been reported by some authors [20,21].

In this study, six plants of the Lamiaceae family (*Stachys tmolea* Boiss.*, Stachys thirkei* C. Koch, *Ballota acetabulosa* (L.) Benth., *Thymus sipthorpii* Benth., *Satureja aintabensis* P.H. Davis and *Micromeria juliana* (L.) Benth. ex Reich.) were investigated with traditional claims for several diseases. The objective of the present study was to evaluate the antimycobacterial activities and determine the phenolic composition of *S. tmolea, S. thirkei, B. acetabulosa, T. siptorpii, S. aintabensis,* and *M. juliana.* To the best of our knowledge, these six plants had not previously been screened for their activity against *M. tuberculosis*.

## Methods

### Plant materials

Six Turkish medicinal plants of the family Lamiaceae (*S. tmolea, S. thirkei, B. acetabulosa, T. sibthorpii, S. aintabensis* and *M. juliana*) were selected for their antimycobacterial activities. All the plant materials were further identified in the Department of Biology, University of Balikesir-Turkey, by Professor Gulendam Tumen. Voucher specimens were deposited at the Herbarium of Balikesir University, Department of Biology. The details herbarium data about plants were given with their locality, altitude, collection time, acquisition code numbers are listed in Table 1.

**Table 1:** Plant herbarium data according to locality, altitude, collection time, and herbarium number

**Plant no**	**Plant species**	**Locality**	**Altitude**	**Collection time**	**Herbarium number**
1	*Stachys tmolea* Boiss.	Balikesir, Edremit, Kazdagi National Park	1400 m	11 June 2010	FS1550
2	*Stachys thirkei* C. Koch.	Sakarya, Akyazı, Davlumbaz plateau	1300 m	13 June 2010	FS1551
3	*Ballota acetabulosa* (L.) Benth	Balikesir Gomec, Madra	300 m	3 June 2010	FS1552
4	*Thymus sibthorpii* Benth.	Tekirdag	1500 m	20 June 2009	FS1553
5	*Satureja aintabensis* P.H. Davis	Gaziantep, Duluk Forest	850 m	15 July 2009	FS1554
6	*Micromeria juliana* (L.) Benth. ex Reich	Balikesir Edremit, Kazdagi	450 m	6 August 2009	FS1555

### Preparation of extracts

The dried aerial parts of each plant (six plants) were cut into small pieces, weighed and subjected to a sequential extraction with different solvents according to their increasing polarity: petroleum ether, ethyl acetate, and methanol. For each sample, the plant material and respective solvents were left to macerate for two weeks at room temperature. The extracts were filtered through Whatman’s No. 1 filter paper. The residue was re-extracted successively with the different solvents mentioned. Solvents were evaporated under reduced pressure using a rotavapor (Heidolph). Therefore, three fraction extracts of petroleum ether fraction (PEF), ethyl acetate fraction (EAF) and methanol fraction (MEF) were obtained for each herb. The total extraction yield, expressed as a percentage, is given in Table 2. Dry fractions were stored in a deepfreeze at −20°C until use.

**Table 2:** Antimycobacterial activity (MIC and MBC, μg/ml) of the 18 plant extracts

**Plant species**	**Solvent**	**Extraction Yields (%)**	**Minimum inhibitory concentration (μg/ml)**				**Minimum bactericidal concentration (μg/ml)**			
			**H37Rv**	**H37Ra**	**Strain 1**	**Strain 2**	**H37Rv**	**H37Ra**	**Strain 1**	**Strain 2**
*Stachy stmolea*	PEF	0,59	>6400	>6400	nd	nd	-	-	-	-
	EAF	3,02	>6400	>6400	nd	nd	-	-	-	-
	MEF	6,52	>6400	>6400	nd	nd	-	-	-	-
*Stachys thirkei*	PEF	0,41	>6400	>6400	nd	nd	-	-	-	-
	EAF	4,87	>6400	>6400	nd	nd	-	-	-	-
	MEF	11,21	>6400	>6400	nd	nd	-	-	-	-
*Ballota acetabulosa*	PEF	0,73	200	400	800	400	800	800	1600	1600
	EAF	2,30	>6400	>6400	nd	nd	-	-	-	-
	MEF	8,87	>6400	>6400	nd	nd	-	-	-	-
*Thymus sibthorpii*	PEF	0,61	12,5	50	800	400	800	800	800	800
	EAF	3,18	12,5	12,5	400	400	400	400	400	400
	MEF	3,85	800	200	1600	800	>6400	>6400	>6400	>6400
*Satureja aintabensis*	PEF	0,58	25	50	100	100	100	100	100	100
	EAF	3,24	12,5	12,5	50	50	50	50	50	50
	MEF	6,39	100	400	3200	1600	>6400	>6400	>6400	>6400
*Micromeria juliana*	PEF	0,29	1600	1600	-	nd*	>6400	>6400	-	-
	EAF	2,32	100	50	200	400	>6400	>6400	>6400	>6400
	PEF	5,26	1600	1600	-	-	>6400	>6400	-	-
Standard drugs										
	SM		0.63	0.63	1.25	0.63	-	-	-	-
	INH		0.16	0.31	0.31	0.31	1.25	1.25	1.25	1.25
	RIF		0.78	0.39	0.78	0.78	6.25	3.13	6.25	6.25
	EMB		2.73	2.73	2.73	1.37	-	-	-	-

*Nd*, not determined due to the fact that the samples were not active on H37Rv and H37Ra; nd*: not determined due to insufficient amount of the sample;(−) not tested; *PEF*, Petroleum ether fraction; *EAF*, Ethyl acetate fraction; *MEF*, Methanol fraction; *SM*, Streptomycin; *INH*, Isoniazid; *RIF*, Rifampicin; *EMB*, Ethambutol.

### Liquid chromatography–mass spectrometry analysis

#### Chemicals and samples

Gradient grade MeOH and acetonitrile were purchased from Merck. Gradient grade water (18 m) was prepared by using a Purelab Option-Q elga dv25 system. All standard stock solutions (1 mg/ml) were prepared by dissolving each compound in MeOH. Standards, rosmarinic acid, trans-cinnamic acid, and ferulic acid were purchased from Aldrich, caffeic acid and gallic acid from Sigma-Aldrich and all other chemicals used were obtained from Sigma. All solutions were passed through a membrane filter (Sartorius, Ø 0.22 μm.) before injection into the capillary.

### Liquid chromatography–mass spectrometry conditions

Analyses were performed with an Agilent Liquid chromatography–mass spectrometry (LC-MS) system (1200 LC with a single quadrupole) with Electrospray Ionisation (ESI) source-negative mode. Source parameters were optimized to provide highest sensitivity. The source parameters are: Gas temperature 350°C, drying gas flow 12 l/min, nebulizer pressure 50 psi, capillary voltage 3500 V. Separation was carried by a Poroshell 120 SB-C18 column (2.1 × 100mm 2.7um). Mobile phases are A: Water (10 mM Ammonium Acetate + 0.2% Formic Acid) and B (100% Methanol). The gradient program is: Start with 10% B and hold 0.2 min; then increased to 30% B until 3 min; increase B to 80% until 20 min; increase B to 95% at 20.1 min and hold 2 min; decrease B to 10%. Total run time is 35 min. Injection volume is 5 μl. The detection was accomplished using MS SIM mode. Scan mode is also used. LC–MS analysis was based in the method described by [22].

### Stock and working solutions

For antimycobacterial activity, stock solutions of extracts were prepared in advance at a concentration of 100 mg/ml in dimethylsulphoxide (DMSO, Merck, Germany) and sterilized by filtration through membrane filter (Ø 0.20 μm; Minisart, Sartorius Bitech, Germany). Stock solutions were kept at −20°C until use. Before the bioassay, working solutions were prepared by diluting stock solutions to four times (4×) the maximum desired final testing concentration in sterile Middlebrook 7H9 broth. The final concentration of DMSO in all assays was ≤2%, and this was used as a solvent control. It has been shown to be nontoxic for mycobacteria at this concentration.

### Anti-tuberculosis drugs

Isoniazid (INH), rifampicin (RIF), streptomycin (SM), and ethambutol (EMB) (Becton, Dickinson and company, Spark, U.S.A) were used as standards. Stock solutions were prepared in sterile distilled water according to the manufacturer's recommendations.

### *Mycobacterium* strains and growth conditions

Two well-characterized strains of *M. tuberculosis* were obtained from the American Type Culture Collection (ATCC): *M. tuberculosis* H37Rv (ATCC 27294) and H37Ra (ATCC 25177), sensitive to all first-line anti-tuberculosis drugs. Two clinical strains namely “strain1” and “strain2” isolated from TB patients were also used. The organisms were maintained on slant of Middlebrook 7H10 agar (Difco, USA) supplemented with 0.5% glycerol and 10% OADC (Oleic acid, Albumin, Dextrose, and Catalase; Difco).

### Preparation of inoculums for anti-tuberculosis assay

All strains were grown at 37°C in Middlebrook 7H9 Broth (7H9) (Becton Dickinson), supplemented with 0.2% v/v glycerol (Sigma Chemical Co., St. Louis, MO, USA), and 10% v/v oleic acid, albumin, dextrose, and catalase (OADC; Becton Dickinson). PANTA 2.5% (Polymixin, Amphotericin B, Naladixic acid, Trimethoprim and Azlocillin; Becton, Dickinson), an antibiotic supplement, was also added to prevent the growth of any non-mycobacteria.

A suspension was prepared in Middlebrook 7H9 Broth. The turbidity of the suspension was adjusted to the McFarland standard 1.0. The suspension was vortexed for several minutes and allowed to precipitate larger particles then to sit for 20 min. The supernatant was transferred to an empty, sterile tube and allowed to sit for a further 15 min. After being transferred to a new sterile tube, the suspension was adjusted to a 0.5 McFarland turbidity standard by visual comparison. One ml of the adjusted suspension was diluted in 4 ml of sterile saline.

To prepare inoculum from a positive BACTEC MGIT tube, the positive tubes were used beginning from the day after it first became positive (day 1 positive) up to and including the fifth day (day 5 positive). The positive tubes older than five days were subcultured into fresh growth-medium. The tubes which were day-1 and day-2 positive proceeded to the inoculation procedure for the susceptibility test. The tubes between day 3 and day 5 positive were diluted using a 1 ml of the positive broth with 4 ml of sterile saline, the total is 5, then this diluted suspension were used for inoculation procedures. Inocula prepared from a Day 1 to Day 5 positive MGIT 7 mL tube range were between 0.8 × 10^5^ to 3.2 × 10^5^ CFU/mL. Each assay was performed according to the MGIT manual Fluorometric susceptibility test procedure recommended by the manufacturer, Becton, Dickinson and Company [23,24].

### Antimycobacterial susceptibility assay

The activity of all extracts against the aforementioned *M. tuberculosis* strains was tested using the Microplate Presto Blue Assay (MPBA) following the procedure previously described Collins & Franzblau [25] and modified by Jimenez-Arellanes et al. [26] using Presto blue reagent. Prior to the bioassay, working solutions of the extracts were prepared by diluting stock extract solutions to four times the maximum desired final testing concentration in sterile 7H9 broth.

### The minimal inhibitory concentration (MIC)

Antimycobacterial susceptibility assay was done according to National Committee for Clinical Laboratory Standards [24,27].

The test was performed in 96-well sterile microplates. All wells received 100 μl of supplemented Middlebrook 7H9 broth. One hundred microliters of a 4× working solution of 18 plant extracts were added to the first column of each row. Using a multichannel pipette, 100 μl was transferred from column 1 to column 2, and the contents of the wells were mixed well. Identical serial 1:2 dilutions were continued through column 10, and 100 μl of excess medium was discarded from the wells in column 10. Then, 100 μl of *M. tuberculosis* inoculum was added to the wells in rows A to H in columns 1 to 11. Thus, the wells in columns 11 and 12 served as drug-free controls. Final test concentrations ranges were 6400–12.5 μg/ml in the mixture.

Each microplate was incubated for 5 days at 37°C. Following incubation, one control growth (well A11) was developed with 20 μl of Presto blue solution (Invitrogen, Life Technologies). The plates were re-incubated at 37°C for 24 h. After this incubation, if the well remained blue, the reagent would be added to another control well and the result would be read on the following day. If the color turned pink, all the wells in microplate received the Presto blue solutions in the same way and were incubated for an additional 24 h and the colors of all wells recorded. A blue color in the well was interpreted as no growth, and wells with a well-defined pink color were scored as positive for growth. The minimal inhibitory concentration (MIC) was defined as the lowest concentration of sample that prevents a color change to pink. Streptomycin, isoniazid, ethambutol and rifampicin were included as standard drugs. Each experiment was performed in triplicate at least twice.

### The minimum bactericidal concentration (MBC)

In this study, bactericidal activity was determined by microdilution methods as described in our recent publication [28]. Briefly, standard broth dilution technique for MICs was first performing as described above. Then, samples of those dilutions with no visible growth were transferred to new plate prepared with fresh supplemented Middlebrook 7H9 broth. Twenty microliters (20 μl) of mycobacterial suspensions were transferred from the former to a new microplate and this was incubated and developed with Presto Blue as for MPBA. The MBC corresponded to the minimum extract concentration that did not cause a color shift in cultures re-incubated in fresh medium [29,30].

## Results and discussion

### LC/MS characterization of the phenolic composition of samples

**Figure 1:**
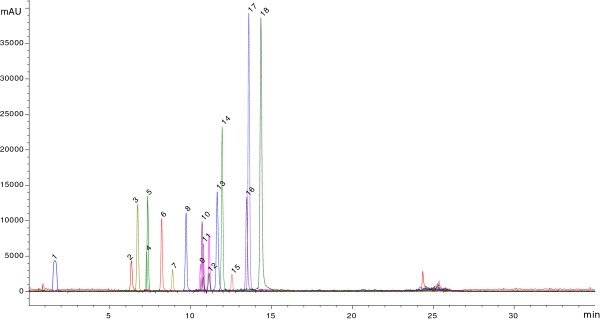
**ESI-TIC-SIM chromatogram of standard phenolics (details according to retention time are given in the Table **3**).**

**Figure 2:**
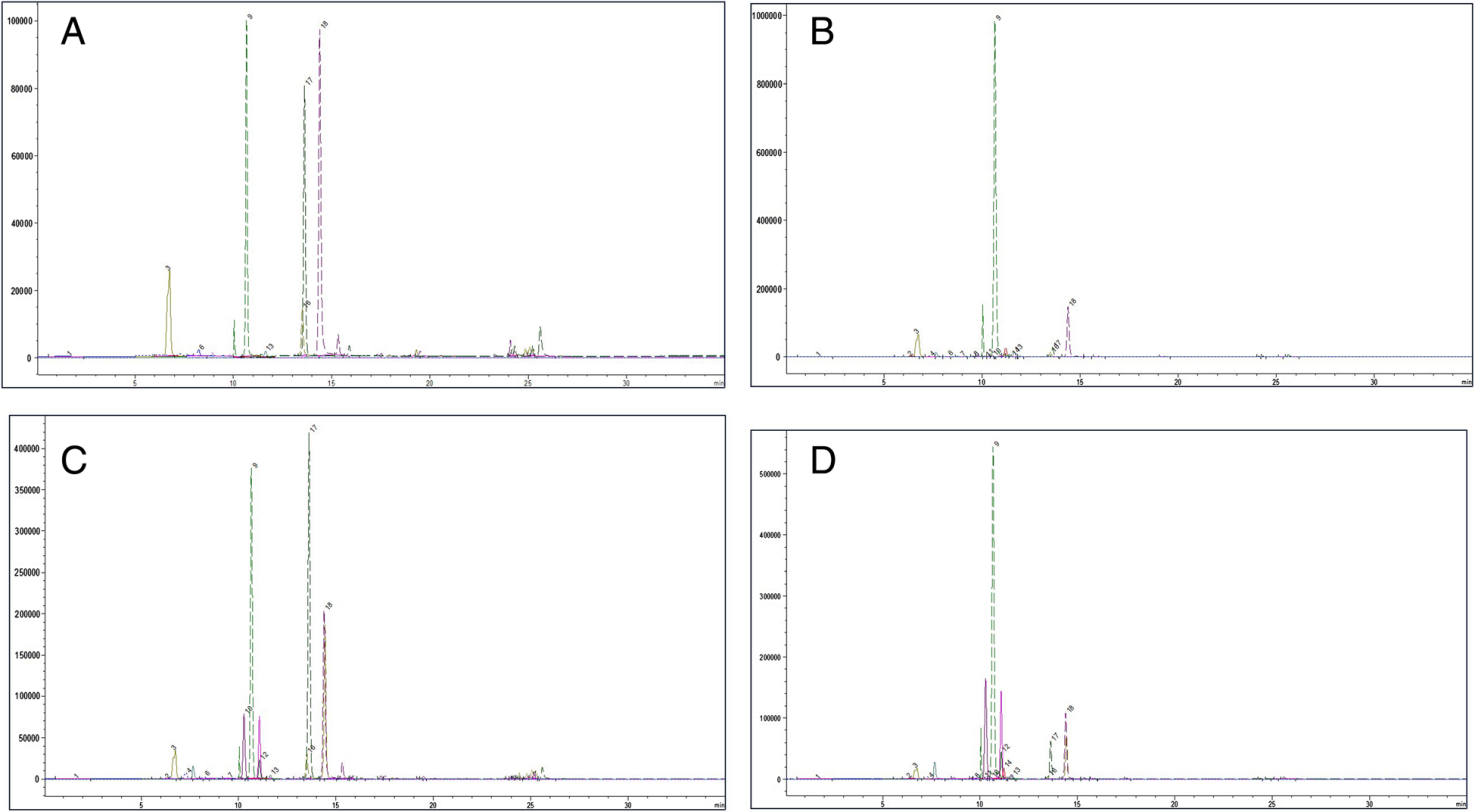
**ESI-TIC SIM chromatogram of A) *T. sibthorpii *** **EAF extract B) *T. sibthorpii *** **MEF extract C) *S. aintabensis *** **EAF extract D) *S. aintabensis *** **MEF extract.**

Standard phenolic, flavonoids and samples were analyzed according to ionization response in ESI mass spectrometry. Their chromatographic retention time were given in Figure 1, Figure 2 and Table 3.The chemical concentrations (μg/g) of phenolic and flavonoids of the tested plants in PEF, EAF and MEF were given in Table 4.

**Table 3:** **LC-MS characteristics of phenolic compounds according to signal no (S.N.), retention times (R.T.) (min), and molecular ions [M-H**^**+**^**](m/z) at fragment ion 80 eV**

**S.N.**	**R.T.**	**[M-H**^**+**^**]**	**Phenolics**	**Samples**
1	1,6	169	Gallic acid	1B, 1C, 2B, 2C, 3B, 3C, 4B, 4C, 5B, 5C, 6B, 6C,
2	6,17	353	Chlorogenic acid	1A,1B, 1C, 2A, 2B, 2C, 3A, 4B, 5B, 5C, 6B, 6C
3	6,53	179	Caffeic acid	1B, 1C, 2B, 2C, 3B, 3C, 4B, 4C, 5B, 5C, 6B,6C
4	7,3	197	Syringic acid	4C, 2B, 2C, 4B, 5B, 5C, 6B, 6C
5	7,34	289	(−)-Epicatechin	3C
6	8,28	163	ρ-coumaric acid	1B, 1C, 2C, 3B, 3C, 4B, 4C, 5B, 6C
7	8,9	193	Ferrulic acid	1C, 2C, 4B, 5B, 6C
8	9,72	431	Vitexin	1C, 2B, 2C, 4B, 5B, 6B, 6C
9	10,6	359	Rosmarinic acid	2B, 2C, 3B, 3C, 4B, 4C, 5B, 5C, 6B, 6C
10	10,69	579	Naringin	1B, 1C, 2B, 2C, 3B, 4B, 5B, 5C, 6B, 6C
11	10,76	609	Rutin hidrat	1B, 1C, 2B, 3B, 3C, 4B, 5B, 6B, 6C
12	11,12	610	Hesperidine	1B, 5B, 5C, 6B, 6C
13	11,65	431	Apigenin 7glikozide	1B, 1C, 2B, 2C, 3B, 3C, 4B, 4C, 5B, 5C, 6B, 6C
14	11,96	539	Oleuropein	1C, 3C, 4B, 5B, 6B, 6C
15	12,57	147	*trans*-cinnamic acid	
16	13,47	301	Quercetin	1C, 4B, 4C, 5B, 5C, 6B, 6C
17	13,613	271	(±)-Naringenin	1B, 1C, 2B, 2C, 3C, 4B, 4C, 5B, 5C, 6C
18	14,36	285	Luteolin	1B, 1C, 2B, 2C, 3B, 3C, 4B, 4C, 5B, 5C, 6B, 6C

**Table 4:** Chemical concentrations (μg/g) of phenolic of the tested plants in petroleum ether (PEF), ethyl acetate (EAF) and methanol (MEF) extract fractions

**Plant No**	**Extracts**	**Chemical concentrations of phenolic**																	
		**1**	**2**	**3**	**4**	**5**	**6**	**7**	**8**	**9**	**10**	**11**	**12**	**13**	**14**	**1**	**16**	**17**	**18**
Stm	PEF (A)	*	35,88	*	*	*	*	*	*	*	*	*	*	*	*	*	*	*	*
	MEF (B)	193,86	19821,5	1235,31	*	*	122,21	*	*	*	194,67	75,76	1690,35	5888,53	*	*	*	54,01	214,67
	EAF (C)	504,15	371,03	3422,58	310,78	*	666,26	257,13	206,38	*	276,64	71,33	*	1986,35	195,20	*	68,44	285,56	1038,67
Sth	PEF (A)	*	0,81	*	*	*	*	*	*	*	*	*	*	*	*	*	*	*	*
	MEF (B)	27,02	17965,90	2092,20	363,51	*	*	*	276,36	460,80	202,86	80,34	*	1851,75	*	*	*	66,17	233,10
	EAF (C)	108,31	12192,68	5208,54	1205,07	*	200,43	257,56	263,55	1151,40	136,21	*	*	1255,18	*	*	*	121,72	465,49
Ba	MEF(B)	18,45	6856,7	1567,83	*	*	144,04	*	*	12258,76	301,17	120,96	*	1104,05	*	*	*	*	120,51
	EAF (C)	63,11	189,34	519,04	*	330,09	160,78	*	*	6885,16	*	1451,65	*	151,11	119,81	*	*	30,54	55,11
Ts	MEF (B)	268,9	263,86	5032,83	340,2	*	235,97	251,6	85,24	81432,13	377,93	191,26	*	940,27	141,95	*	645,37	452,04	3632,13
	EAF (C)	39,15	*	1908,9	*	*	148,3	*	*	6074,77	*	*	*	97,8	*	*	666,37	1436,47	2390,07
Sa	MEF (B)	53,45	234,71	1383,2	443,75	*	*	*	106,01	42936,05	260,38	238,73	10117,43	389,33	551,37	*	249,27	1084,98	2446,5
	EAF (C)	93,78	56,14	2587,51	956,49	*	230,23	177,14	*	26995,81	7748,81	*	5633	282,47	*	*	1518,12	7729,86	5025,99
Mj	PEF (A)	*	*	*	*	*	*	*	*	*	*	*	*	*	*	*	*	*	*
	MEF (B)	76,51	5256,91	1603,05	128,17	*	*	*	383,28	32457,86	581	5336,78	6013,16	480,88	201,9	*	684,28	*	242,2
	EAF (C)	129,07	167,96	1434,85	215,8	*	92,85	170,23	550,55	4449,22	60,7	679,12	734,01	92,31	34,1	*	192,37	79,92	226,36

Stm: Stachys tmolea; Sth: Stachys thirkei Ba: Ballota acetabulosa Ts:Thymus sibthorpii Sa: Satureja aintabensis Mj:Micromeria juliana;EAF: EthylAcetat; MEF: Methanol; 1; Gallicacid 2; Chlorogenicacid 3; Caffeicacid 4; Syringicacid 5; Epicatechin 6; P-coumaricacid 7; Ferrulicacid 8; Vitexin 9; Rosmarinicacid 10; Naringin 11; RutinHidrat 12; Hesperidine 13; Apigenin 7 Glikozide 14; Oleuropein 15; Trans-cinnamicacid 16; Quercetin 17; Naringenin 18; Luteolin, *: Not detected

In the evaluation of the phenolic and flavonoid composition of samples; for MEF extracts of *T. sibthorpii;* rosmarinic acid, caffeic acid, apigenin 7 glikozide, quercetin, and luteolin were major phenolic compounds in the sort descending. On the other hand, we also determined gallic acid; chlorogenic acid, *s*yringic acid, p-coumaric acid; ferrulic acid, vitexin, naringin, rutin hidrat, oleuropein and naringenin as minor level.

Epicatechin, hesperidine, and trans-cinnamic acid were not detected. In the EAF extracts of *T. sibthorpii,* rosmarinic acid, luteolin, caffeic acid, naringenin, and quercetin were the major constituents in decreasing concentrations respectively. Gallic acid, p-coumaric,acid and apigenin 7 glycoside were found in less amount. Some of the phenolic and flavonoids such as chlorogenic acid, syringic acid, epicatechin, ferrulic acid, vitexin, naringin, and rutin hidrat were not detected in the EAF extracts of *T. sibthorpii.*

For *S. aintabensis,* while the major phenolic compounds were rosmarinic acid, hesperidin, luteolin, caffeic acid, and naringenin for MEF extract in descending order, gallic acid, chlorogenic acid, syringic acid, vitexin, naringin, rutin hidrat, apigenin 7 glycoside, and quercetin were exist in the moderate to less amount. On the other hand, epicatechin, p-coumaric acid, ferrulic acid, and trans-cinnamic acid were not detected.

In EAF extract of *S. aintabensis;* rosmarinic acid has the highest concentration. It is followed by naringin, naringenin, hesperidine, caffeic acid, and quercetin*.* The lesser quantity of substances were gallic acid, chlorogenic acid, syringic acid, p-coumaric acid, ferrulic acid, and apigenin 7 glycoside. Some of the phenolic and flavonoids such as epicatechin, vitexin, rutin hidrat, oleuropein, and trans-cinnamic acid were not found.

For others, the major phenolic compounds for *M. juliana* were rosmarinic acid, chlorogenic acid, rutin hydrate and caffeic acid in the MEF extract. The major phenolic compound for EAF and MEF extracts of *B. acetabulosa* was chlorogenic acid.

The major phenolic compounds for *S. thirkei* were chlorogenic acid, caffeic acid, ferulic acid, and rosmarinic acid for EAH extract; chlorogenic acid and caffeic acid for MEF extract. For *S. tmolea* the major phenolic compound for EAF was caffeic acid.

The determination of oleuropein in some extracts of plants such as EAF of *S tmolea* and *B. acetabulosa;* MEF of *T. sibthorpii* and *S. aintabensis;* and also EAF and MEF of *M. juliana* was one of the interesting and amazing results for us. Because oleuropein which may have a role in different mechanisms such as antioxidant, signaling mecanism or decreased rates of cancer, commonly presented as related with olive leaves which is high amounts in olive leaves such as 60–90 mg/g of dry matter [31] but only little in olive oil [32].

### Antimycobacterial activity

In this study, LC-MS revealed large quantity of rosmarinic acid in all EAF and MEF extracts that showed antimycobacterial activity: *T. sibthorpii, S. aintabensis, M. juliana*. Many studies have suggested that high level of rosmarinic acid might be associated with antimycobacterial activity [21]. Many biological activities have been described for rosmarinic acid, of which the main ones are: astringent, antioxidative, anti-inflammatory, antimutagen, antibacterial and antiviral [33]. The latter activity is used in the therapy of Herpes simplex infections, with extracts of *Melissa officinalis* containing rosmarinic acid.

As many different methods are available to evaluate anti-tuberculosis activity, no specific cut-off value has yet been established for the anti-tuberculosis activity of plant extracts. In previous studies, plants extracts were considered active against *M. tuberculosis* and other related *Mycobacterium* strains if MIC was ≤100 μg/ml [34]; ≤125 μg/ml [35]; ≤200 μg/ml [26,36]; ≤500 μg/ml [37]; ≤1600 μg/ml [38]; and ≤2048 μg/ml [28]. Other studies considered other quantifiable inhibitions as indication of activity [21,39]. In this study, we interpreted activity as inhibition at any values of MIC ≤6400 μg/ml, the highest test concentration. Plant extracts that did not exhibit inhibition at up to 6400 μg/ml were considered inactive. However, activity was classified as significant if MIC < 100 μg/ml, moderate if 100 < MIC ≤ 625 μg/ml or weak if MIC > 625 μg/ml.

Eighteen crude extracts from 6 Turkish plant species of the family Lamiaceae were obtained by successive extraction with three solvents of varying polarity.

The result showed that methanol gave highest yield of extract compared to petroleum ether and ethyl acetate extraction; the yield from petroleum ether was very low (Table 2).

*M. tuberculosis* H37Rv ATCC 27294 and *M. tuberculosis* H37Ra ATCC 25177 variants were used as target organisms in the preliminary assays; all plant extracts that exhibited activity were further tested against two clinical strains (strain 1 and strain 2). The results are shown in Table 2.

Our rationale for using H37Rv (ATCC 27294) and H37Ra (ATCC 25177) was that they have drug susceptibility profiles that are reasonably representative of the majority of drug-susceptible clinical isolates [40]; In addition, they are used around the world as a standard, and could provide an opportunity to identify new compounds effective against strains of *M. tuberculosis*. These results showed that 10 of 18 extracts obtained from three solvents tested, exhibited anti-tuberculosis activity against all the tested *M. tuberculosis* strains, with MICs in the range 12.5–1600 μg/ml and MBC 50–1600 μg/ml. Out of these, according to the classification used [41]: The three fractions of *S. aintabensis* exhibited significant activity. The PEF and EAF of *S. aintabensis* exhibited the most potent activity against all the tested mycobacteria, with MICs value of 12.5–100 μg/ml, while the activity of its MEF was significant against *M. tuberculosis* H37Rv (MIC 100μg/ml, moderate against H37Ra (MIC 400 μg/ml) and weak against the two clinical strains. PEF and EAF of *S. aintabensis* not only inhibited the growth of mycobacteria, but also showed bactericidal effect, with MBC value of 50 and 100 μg/ml against all the tested organisms. It is known that phenolics are effective antimicrobial agents against a wide array of microorganisms [42]. The strong antimycobacterial properties of *S. aintabensis* observed in the present study could be due to the presence of phenolic compounds such as rosmarinic acid, naringenin, hesperidin, luteolin and caffeic acid. This is the first study of the anti-tuberculosis activity and chemical composition of *S. aintabensis.* Antimycobacterial activity of *S. aintabensis* was not previously reported.

The two fractions of *T. sibthorpii*, PEF and EAF, exhibited significant activity against H37Rv and H37Ra with MICs value of 12.5–50 μg/ml; and moderate activity against the two clinical strains (MIC 400 μg/ml). The MEF of *T. sibthorpii* exhibited moderate activity against H37Ra (MIC 200 μg/ml) and weak activity against the three others strains (800 and 1600 μg/ml). The PEF and EAF of *T. sibthorpii* also showed bactericidal effect against all the tested respectively *Mycobacteria*, with MBC values of 400 and 800 μg/ml.

The good antimycobacterial properties of *T. sibthorpii* could depend on the presence of phenolic compounds such as rosmarinic acid and caffeic acid. *T. sibthorpii* was previously screened for its antimicrobial activity, and its ethanol extract was reported to show broad antimicrobial activity spectrum. Recent studies have shown that *Thymus* species have strong antibacterial, antifungal [43], antiviral and expectorating [44], activities, but no data on its antimycobacterial activity seem to have been published previously.

The PEF of *B. acetabulosa* presented moderate activity against H37Rv, H37Ra and clinical strain 1 (MIC 200–400 μg/ml), and weak activity against clinical strain 2 (MIC 800 μg/ml). This extract was also bactericidal, with MBC value of 800 μg/ml against H37Rv and H37Ra and 1600 μg/ml against the two clinical strains. The EAF and MEF of *B. acetabulosa* did not show any activity against the tested strain Plants of this genus have been traditionally used to treat nausea, vomiting, nervous dyspepsia, and also are used for their antiemetic, sedative, antibacterial and mild astringent properties [45]. Aerial parts of the plant are used internally to treat inflammation, to suppress cough and against gastrointestinal disorders [46]. The antibacterial and antifungal activities of *B. acetabulosa* were reported by Dulger and Sener [46]. The antioxidant activity of *B. acetabulosa* has been reported [10], but to the best of our knowledge, the antimycobacterial properties have not been reported previously.

The EAF of *M. juliana* showed a most potent activity with the minimal inhibitory concentration (MIC) value of 100 and 50 μg/ml, while the corresponding PEF and MEF exhibited very weak activity (MIC 1600 μg/ml each). *M. Juliana* extracts have no bactericidal effect. The antimycobacterial activity exhibited by *M. juliana* probably depends on the quantity of phenolics such as rosmarinic acid, chlorogenic acid, rutin hydrate and caffeic acid.

It is also noticeable that all plant extracts containing high levels of chlorogenic acid were inactive or showed very weak activity against the strain of *M. tuberculosis*. This is the case for MEF of *M. juliana*, MEF of *B. acetabulosa*, MEF and EAF of *S. thirkei*, and EAF of *S. tmolea*.

The large amount of chlorogenic acid could react as an antagonist to inhibit the activity of other compounds like rosmarinic acid. When the composition of *T. sibthorpii* and *S. aintabensis* extracts are compared to *M. Juliana* extract, the three plants contain a large amount of rosmarinic acid, however, *T. sibthorpii* and *S. aintabensis* contain very low levels of chlorogenic acid, whereas *M. juliana* is very rich in chlorogenic acid.

The two *Stachys* species (*S. tmolea* and *S. thirkei*) showed no activity against the screened strains in our assays. Essential oil from *S. tmolea* was investigated by disk diffusion method on *C. albicans* by Goren et al. (2011) [47] and was reported to show antifungal activity. To the best of our knowledge, the above-mentioned antifungal activity was the only previous reported activity on *S. tmolea.* Regarding *S. thirkei*, no activity has been evaluated until now.

The finding that the major phenolic compound of *S. tmolea* and *S. thirkei* is chlorogenic acid could partially explain their inactivity against *M. tuberculosis*.

In this study, we reported that PEF and EAF were most active compared to the methanol fraction. This might be because the mycobacteria, having a very lipid-rich hydrophobic cell-wall, are often more susceptible to compounds with weaker polarity [48].

## Conclusion

Tuberculosis is a very serious disease in the world. A total of 1.4 million people died from TB in 2011. One third of the world’s population, especially from low- and middle-income countries, is infected with *Mycobacterium tuberculosis*. In addition, approximately one in four deaths among people with HIV is due to TB. MDR-TB, affecting 630,000 people in the world, is caused by inappropriate use of TB-drugs, does not respond to standard treatments because it is costly and ineffective to treat [49].

In the present investigation, we also demonstrated for the first time the efficacy of the PEF and EAF of two plant species*, S. aintabensis* and *T. sibthorpii*. In addition, PEF extract of *Ballota acetabulosa* was also efficient against *M. tuberculosis* strains including isolates taken from patients. These extracts displayed very high activity, not only by inhibiting but also by killing all of the *Mycobacteria* strains. The phenolics and flavonoids might represent promising sources of anti-TB drugs. The LC-MS spectra of all the extracts revealed their main phenolic composition, which was closely associated with the observed antimycobacterial activity [50]. However, advanced research will be necessary to confirm this hypothesis.

Consequently, compounds in the extracts and potential activity may guide the future isolation and antimycobacterial evaluation of the active principles. Further phytochemical and pharmacological studies of these plants are clearly worthwhile. Flavonoids might be “a modal” for the drug design. Identification of the antimycobacterial mechanism of action might be the key to the development of these compounds.

## Abbreviations

TB: Tuberculosis; MDR: Multi-drug resistance; SLD: Second-line drug; DOTS: Directly observed treatment short course; HIV: Human immunodeficiency virus; Rt: Retention time; ATCC: American type culture collection; OADC: Oleic acid albumin, dextrose, and catalase; PANTA: Polymixin amphotericin B, naladixic acid, trimethoprim and azlocillin; MGIT: Mycobacteria growth indicator tube; BACTEC: Battle area clearance training, equipment and consultancy; INH: Isoniazid; RIF: Rifampicin; SM: Streptomycin; EMB: Ethambutol; DMSO: Dimethyl-sulphoxide; ESI: Electrospray ionisation; MeOH: Methanol; PEF: Petroleum ether fraction; EAF: Ethyl acetate fraction; MEF: Methanol fraction; AST: Antimicrobial susceptibility testing; MIC: Minimal inhibitory concentration; MBC: Minimal bactericide concentration; LC-MS: Liquid chromatography–mass spectrometry; MPBA: Microplate prestoblue alamar assay; ESI: Electrospray ionisation; TIC: Total ion current; SIM: Selected ion monitoring.

## Competing interest

The authors declare that they have no competing interest.

## Authors’ contributions

EMT carried out the experimental part such as preparation of plant extracts, preparation of inoculums, evaluation of antimycobacterial activity, preparation of sample for LC-MS and manuscript writing. TA provided the mycobacteria strains, supervised the work, evaluated the results and corrected the manuscript for publication. FS provided some plants and corrected the manuscript for publication. SM contributed to antimycobacterial evaluation and LC-MS analysis. HA contributed to LC-MS analysis. All authors read and approved the final manuscript.

## Pre-publication history

The pre-publication history for this paper can be accessed here:

http://www.biomedcentral.com/1472-6882/13/365/prepub
